# Radiographic Study of Transcrestal Sinus Floor Elevation Using Osseodensification Technique with Graft Material: A Pilot Study

**DOI:** 10.3390/biomimetics9050276

**Published:** 2024-05-04

**Authors:** Khrystyna Sulyhan-Sulyhan, Javier Barberá-Millán, Carolina Larrazábal-Morón, Julián Espinosa-Giménez, María Dolores Gómez-Adrián

**Affiliations:** 1 Department of Dentistry, Faculty of Medicine and Health Sciences, Catholic University of Valencia San Vicente Mártir, 46001 Valencia, Spain; khrystyna.sulyhan@ucv.es (K.S.-S.); carolina.larrazabal@ucv.es (C.L.-M.); julian.espinosa@ucv.es (J.E.-G.); mariadolores.gomez@ucv.es (M.D.G.-A.); 2 Doctoral School, Catholic University of Valencia San Vicente Mártir, 46001 Valencia, Spain

**Keywords:** atrophic maxilla, sinus augmentation, bone regeneration, dental implant, maxillary sinus, minimally invasive, surgical procedure, bone substitutes

## Abstract

This pilot study aimed to evaluate the level of implant success after transcrestal sinus floor elevation (tSFE) using the osseodensification technique (OD) combined with beta-tricalcium phosphate (β-TCP) by analyzing clinical and radiographic results. Moreover, the increase in bone height was analyzed immediately after surgery, 3 months after, and before loading by taking standardized radiographic measurements. Thirteen patients, four males and nine females, with a mean age of 54.69 ± 5.86 years, requiring the placement of one implant in the upper posterior maxilla, with a residual bone height of <8 mm and a minimum bone width of 5 mm, participated in the study. The bone gain data was obtained using cone-beam computed tomography (CBCT) immediately after surgery and twelve months after the placement. The correlation between initial and final bone height with implant stability was also assessed. The results were analyzed using SPSS 23 software (*p* < 0.05). The results of the study indicated a 100% implant success rate after a follow-up period of twelve months. Preoperative main bone height was 5.70 ± 0.95 mm. The osseodensification technique allowed a significant increase of 6.65 ± 1.06 mm immediately after surgery. After a twelve-month follow-up, a graft material contraction of 0.90 ± 0.49 mm was observed. No correlation was observed between the bone height at the different times of the study and the primary stability of the implant. Considering the limitations of the size sample of this study, the osseodensification technique used for transcrestal sinus lift with the additional bone graft material (β-TCP) may provide a predictable elevation of the maxillary sinus floor, allowing simultaneous implant insertion with adequate stability irrespective of bone height limitations.

## 1. Introduction

The posterior maxilla represents a clinical challenge due to the lack of bone volume and anatomical limitations that have been overcome by the development of different techniques and a large number of innovative surgical procedures [1,2,3].

Maxillary sinus floor elevation with a lateral (lSFE) or transcrestal (tSFE) approach represent two surgical options to vertically enhance the available bone in the edentulous posterior maxilla [4,5,6]. The osteotome sinus floor elevation (OSFE) technique is a less invasive approach, is less time-consuming, and reduces postoperative discomfort by using osteotomes to increase the density of the maxillary bone by compaction and allowing the insertion of the implants with good primary stability [4]. To reduce trauma and risk of membrane perforation, which is the most common intraoperative complication associated with maxillary sinus lift surgery [7,8], several techniques for the transcrestal approach have been discussed [9,10], including balloon elevation technique [11], hydraulic sinus condensing technique [12], trephine assisted lifting, MISE technique, and CAS KIT (NeoBiotech, quokkaMED O.E. Athens, Greece) [13]. Other advantages of the transcrestal approach are the possibility of combining the sinus elevation and regeneration, with biomaterials or without biomaterials, with the simultaneous insertion of the implants in one surgical step [9]. However, the tSFE technique may present some disadvantages associated with a lower level of bone gain and not having direct optical control, and it is a technique that requires a minimal residual bone height in order for it to be performed [14,15].

Regarding this last factor, to date, there is sufficient scientific evidence demonstrating that the technique is safe and successful with 5 mm of residual bone high (RBH) [16,17,18]. Using this technique, the reconstruction of the posterior maxilla can be simplified and accelerated. No evidence has indicated a critical threshold RBH value for the survival of implants placed with OSFE [18].

In order to obtain greater bone gain during sinus augmentation procedures, different graft materials mixed with or without autologous bone have been frequently used [19,20]. Autogenous bone grafts are considered the gold standard, however, to reduce the morbidity of the donor area, tricalcium phosphate was the first bone substitute to be successfully applied for sinus floor elevation and some studies have registered the improvement in the postoperative period; limited pain and fewer complications were noted [21]. Nevertheless, the necessity of grafting material to maintain the space for new bone formation after elevating the sinus membrane by using the crestal approach remains controversial [22].

As the residual bone height of the maxillary sinus determines the surgical augmentation technique, the hypotheses that have been tested in this study are the possible correlation between the bone height during different moments of the study with insertion torque and primary stability. 

The study primarily aimed to evaluate implant success variations after transcrestal sinus floor elevation (tSFE) using the osseodensification technique (OD) combined with beta-tricalcium phosphate (β-TCP) by analyzing clinical and radiographic results. Moreover, the increase in bone height was analyzed immediately after surgery, 3 months after surgery, and before the implant loading by taking standardized radiographic measurements. 

Secondarily, the study evaluated if there was any correlation between the RBH, the insertion torque, and the ISQ obtained at the time of implant placement, as recent studies consider reduced RHB as a potential risk factor for implant failure due to lack of primary stability [23].

## 2. Materials and Methods

### 2.1. Study Design

The study was designed as a prospective pilot clinical trial and approved by the ethics committee of the Catholic University of Valencia San Vicente Mártir (Valencia, Spain) (Expte.UCV/2019–2020/040). All the clinical procedures were performed in full accordance with the Declaration of Helsinki 2013, Fortaleza version, and CIOMS, 2002. Each patient provided written informed consent before participation. This manuscript was prepared in full accordance with Organic Law 3/2018 on the Protection of Personal Data and ensuring compliance with the regulations.

Patients were consecutively recruited and treated at the Catholic University of Valencia San Vicente Mártir from May 2020 to May 2021. Surgical procedures were performed by students in the second year of their Master of Oral Surgery and Implantology degree, all having similar surgical experience, under the supervision of experienced clinical professors during the period of the study.

Inclusion criteria for patient eligibility were as follows: (i) age ≥ 18 years; (ii) systemic and local conditions suitable for implant placement and sinus floor elevation procedures and non-smoking patients or those smoking ≤10 cigarettes/day; (iii) indication for the placement of at least one implant in premolar or molar region of the maxilla with insufficient bone height (<8 mm), without the need for horizontal bone augmentation (minimum width of 5 mm); (iv) patient willing and fully capable of complying with the study protocol; and (v) minimum follow-up of one year.

Exclusion criteria were as follows: (i) any general contraindication for implant surgery (i.e., uncontrolled diabetes or hypertension, acute or chronic rhinosinusitis/sinusitis, and allergic rhinitis); (ii) localized contraindications for implant surgery: acute or chronic infection, inflammation in the area intended for implant placement, and tooth extraction < 3 months after surgery; (iii) untreated periodontal disease or poor oral hygiene and non-collaborators (plaque index > 25%); and (iv) patients with an INR (international normalized ratio) outside the range of 2–4.

### 2.2. Statistical Analysis 

To obtain the sample, consecutive non-probabilistic sampling was used, and the bone availability was studied before surgery and immediately after surgery, and the final bone height assessed at 12 months. The standard deviation was obtained from the study by Gonzalez et al. [24]: 0.63, and the difference between the initial and final mean was 0.55. A sample size of 13 patients was estimated to ensure that the sample data came from a population with a normal distribution. To study whether the differences were statistically significant, we used comparison *t*-tests for related samples. Moreover, a bivariate correlation test was performed, and since the sample size is small, the Spearman correlation test was also used.

The statistical analysis of the data collected for the present study was performed using the SPSS 23 computer program, using a confidence level of 95% and considering statistically significant these comparison results for which the *p*-value obtained was less than 0.05. 

### 2.3. Radiographic Study

All included patients underwent a full-mouth screening; restorative, endodontic, and periodontal treatments were performed as needed. For each patient, cone-beam computed tomography (CBCT) (Galileo system, Dentsply Sirona^®^ Charlotte, NC, USA) imaging was performed preoperatively (T0), and immediately after surgery (T1) they underwent a CBCT. A preoperative CBCT T0 was taken to identify the location of the sinus, the presence of any septum sinus, possible sinusitis, assess the alveolar-antral artery, and accurately study the amount of bone available. Moreover, the bite height was registered to standardize all measurements. Radiographic measurements of bone height and width were obtained at T1 and T2. Radiographic measurements of bone height and width were obtained by a blinded operator at each stage.

### 2.4. Surgical Procedure

**Figure 1:**
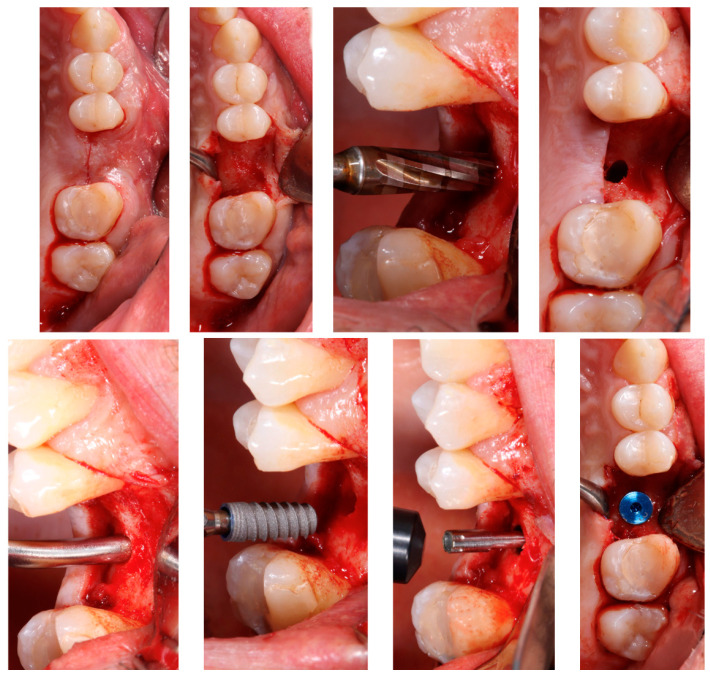
First line, from left to right: Incision; flap elevation; Densah^®^Bur (Jackson, MI, USA); and osteotomy. Second line, from left to right: application of Novabone^®^ graft (LLC Alachua, FL, USA); implant insertion; ISQ measurement; and implant placement.

Surgical procedures were performed by second-year students on the Master of Oral Surgery and Implantology degree course, and involved five different clinicians, all of them with similar surgical experience, supervised by a professor expert in the OD and tSFE. Forty-eight hours before the surgery, a course of antibiotic prophylaxis was introduced by prescribing Amoxicillin/Clavulanic Acid (875/125 mg, tablets) with a regimen of 1 pill every 8 h for 1 week. In the case of those allergic to penicillin, this was replaced by Azithromycin (500 mg, tablets) with a regimen of 1 pill every 24 h for 3 days. After local anesthesia (4% articaine with epinephrine 1:100,000), full-thickness flaps were reflected following a crestal incision and vertical releasing incisions, if necessary. The preparation of the implant site/s was performed with Densah^®^ burs by the Versah^®^ system (Jackson, MI, USA), which allows the compaction of the bone by small increments and a smooth expansion of the osteotomy, alternating between VT5 and VT8 (Figure 1) with abundant irrigation. Drills were rotated in a reversed, non-cutting direction allowing alveolar bone preservation and an increase in bone density. 

The implant site preparation was started with a pilot drill to perforate the cortical bone, following the producer’s instructions, drilling at 1200 rpm clockwise. Then a second drill (2.0) was used, drilling at 1200 rpm counterclockwise to define the orientation of the implant, to the depth of 1–1.5 mm away from the sinus floor. The final diameter of the osteotomy preparation had an average diameter of between 0.5 and 0.7 mm less than the diameter of the implant. All drills from the pilot were used at 1200 rpm in a counterclockwise direction with great irrigation until the graft procedure began. Grafting material, beta-tricalcium phosphate (ß-TCP) NovaBone^®^ Dental Putty (Novabone Products, LLC Alachua, FL, USA), was applied in the alveolar bed in a gel format and compacted by the last drill used to break the sinus floor, which was rotated in a reversed direction, at 50 rpm, and without irrigation. While compacting, for each grafting injection the drilled was inserted 1–1.5 mm deeper. An attempt to standardize the quantity of graft material placed into the sinus was made using the contents of one of the packages (i.e., about 0.25–0.50 cc). All the implants (Klockner^®^ Vega, Soadco, Escaldes-Engordany, Andorra) were submerged and covered with soft tissue. The implants obtained an average insertion torque of 37.23 Ncm^3^ (NSK, Nakanishi Inc., Kyoto, Japan) and an average ISQ of 73.23 (Penguin^®^ Integration Diagnostics Sweeden AB, Gothenburg, Sweden). All implants were left submerged and primary closure of the wound was obtained. 

### 2.5. Postoperative Care

All subjects received appropriate postoperative instructions and continued with the antibiotics prescribed, as well as taking analgesics (paracetamol 1 g or ibuprofen 400 mg every 6 h, if needed). The suture was removed after 15 days. 

### 2.6. Data Collection

The following main variables (dependent variables) were registered: residual bone height (RBH); apical bone gain (ABG); residual bone width (RBW); implant insertion torque (IT); and resonance frequency analysis (RFA). The analysis of secondary variables (independent variables) was fundamental for the evaluation of the efficacy of tSFE with OD and for determining the survival rate. Secondary variables such as age, sex, location of the implant, bone type, and drilling protocol were also registered. 

Among the many factors mentioned, the ISQ value (implant stability coefficient) constitutes the mean unit of resonant frequency analysis (RFA). This parameter is crucial for the osseointegration of the implant. It is a mechanical process which depends on the characteristics of the bone where it is anchored. It is the result of the compressive stress generated into the bone during the insertion of the implant; in other words, the insertion torque of the implant is the measure of the frictional resistance that the implant encounters as it advances apically by means of a rotary movement on its axis. Therefore, this method provides information on the quality of the bone at the implant placement site and the primary stability of the implant.

**Figure 2:**
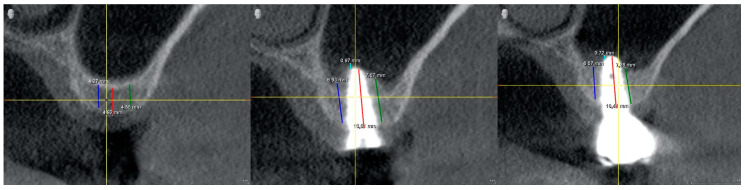
Cross-sections of the CBCT with the measurements made at different moments of the study (T0, T1, and T2).

In the radiographic study, the following parameters were recorded: RBH: residual bone/available bone height, measured parallel to the implant axis, from the bone crest to the sinus floor; RBW: residual bone width, measured in the vestibular-palatal direction; and ABG: apical bone gain. Distance from implant apex-most apical bone was measured along the axis of the implant. This value was considered to be zero if the Schneiderian membrane was left in contact with the apex of the implant. All CBCT cross-sections were collected and analyzed by the same calibrated (intraclass correlation coefficient) and blinded doctor, and measurements were made by taking landmarks of the antagonist quadrant and adjacent structures (Figure 2). 

## 3. Results

### 3.1. Study Population 

Sixteen implants were initially included in the study, however, three patients dropped out because they could not attend programmed follow-up reviews. Therefore, thirteen patients and implants were finally included in the study (nine implants of 10 mm length and three of 8 mm). Regarding the demographic characterist ics, four males and nine females participated in the study, with a mean age of 54.69 ± 5.86 years (Table 1). Demographic characteristics are reported in Table 1. The implant survival rate was 100% because none of the implants failed. The description of aspects related to the surgical procedure is shown in detail in Table 2. 

### 3.2. Surgical and Postsurgical Complications

In this study, perforations of the Scheneiderian membrane were not detected, which was verified by the immediate postoperative CBCT. The overall procedure was well tolerated by the patients, who complained of only slight postoperative swelling and minor discomfort. During the first postoperative week, one patient reported nose bleeding.

### 3.3. Analysis of the Variation in Residual Bone Height 

**Figure 3:**
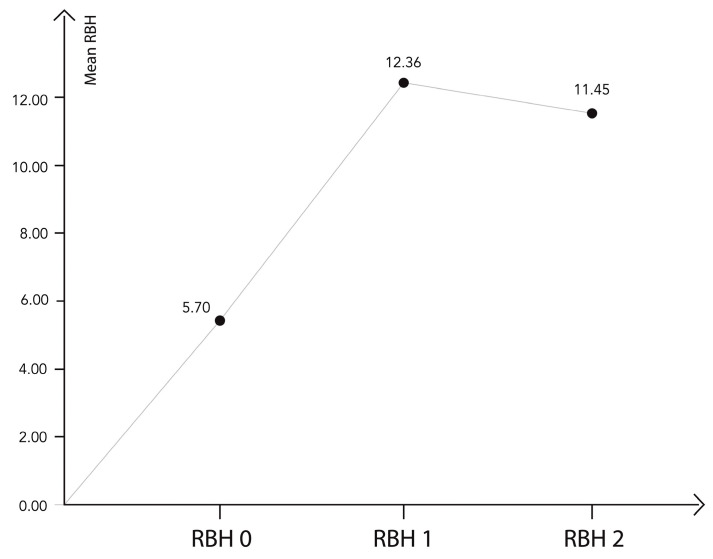
Distribution of the main variable, the residual bone height at different times of the study. There was a significant increase between the preoperative moment (T0) and moments T1 and T2, and a significant decrease between moments T1 and T2.

The mean RBH preoperatively (T0) was 5.70 ± 0.95 mm (IR: 3.47 to 8.40), immediately after surgery RBH (T1) was 12.36 ± 1.20 mm, and after a 12-month follow-up RBH (T2) was 11.45 ± 0.94 mm (Figure 3). The results of RBH variations are shown in Table 3.

**Figure 4:**
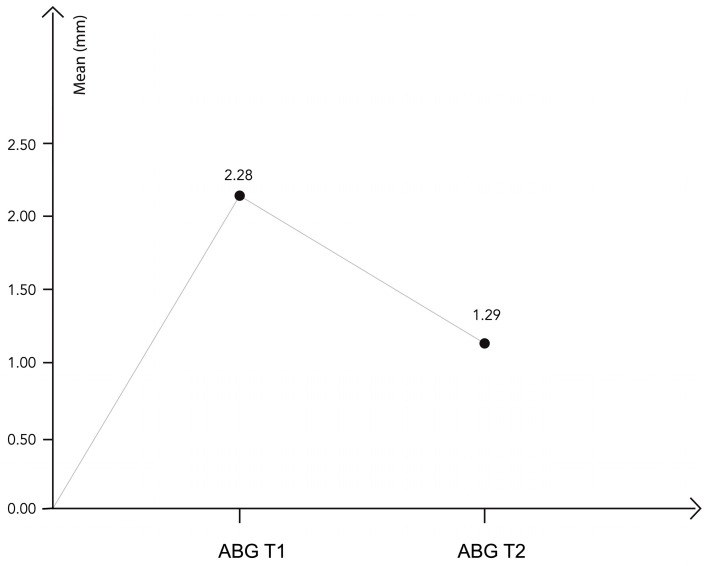
The apical bone gain at different times of the study.

A mean considerable increase of 6.65 ± 1.06 mm was achieved immediately after transcrestal sinus lift surgery (T0–T1). A limited physiological contraction of the bone graft (0.90 ± 0.49 mm) was observed during the healing period (T1–T2), as shown in Figure 4. As the sample follows a normal distribution, a comparison *t*-test for related samples was used to analyze these differences, as shown in Table 4.

### 3.4. Comparative Study of the Apical Bone Gain

Apical bone gain (ABG) was studied at each moment of the study (Figure 4) and differences during T1 and T2 were analyzed (Table 5).

The *p*-value of the contrast statistic of the *t*-test was <0.05, thus, the decline in apical bone gain observed between T1 and T2 was statistically significant. There was a significant increase between the preoperative moment (T0) and moments T1 and T2, and a significant decrease between moments T1 and T2 (Table 6).

### 3.5. Study of the Relationship between Primary Stability Parameters and the Available Bone Height at Different Times of the Study

Regarding primary stability parameters, analysis of implant insertion torque (IT) and resonance frequency analysis (RFA), when using the osseodensification technique for transcrestal sinus lift, indicated that there was no statistical evidence for an association between primary stability and the bone height available at the different times of the study (Table 7). 

**Figure 5:**
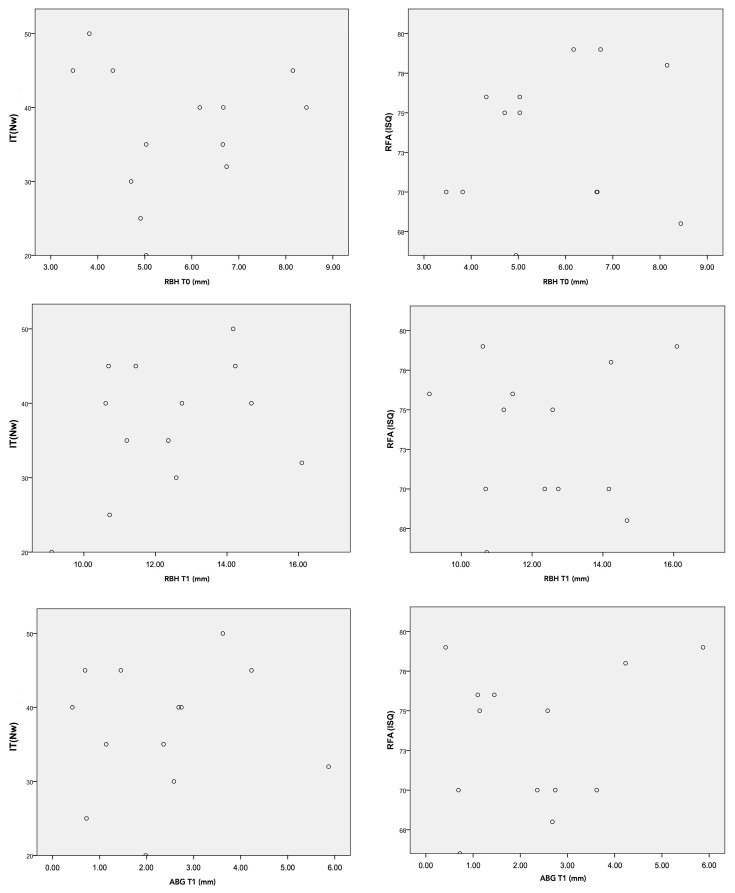
Graphic distribution of the data: relationship between the available bone height and primary stability parameters (RFA and IT).

The *p*-values of the Spearman correlation test were all higher than 0.05, so we did not find statistical evidence to say that there was a correlation between the primary stability parameters (IT–RFA) and the bone height at moments T1 and T2 or the apical bone gain at moment T1. (Figure 5). 

## 4. Discussion

The present study was performed to evaluate implant success and radiographic outcomes of bone height variations after tSFE was performed with the OD technique [25] combined with β-TCP and simultaneous implant placement. This study was conducted as a pilot study where thirteen implants were placed in ten patients consecutively treated with tSFE using the OD technique. This sample is equivalent to other clinical studies focused on tSFE [16,26,27].

Radiograph measurements were performed before surgery (T0), immediately after surgery (T1), and at 12 months (T2). The results of the study showed (i) a 100% implant survival rate after a follow-up period of twelve months, (ii) that the tSFE using OD technique combined with β-TCP resulted in substantial RBH immediately after surgery and at twelve months, (iii) a graft material contraction was observed after a twelve-month follow-up, and (iv) no correlation was observed between the bone height at the different times of the study and the primary stability. 

To rehabilitate the upper posterior maxilla, maxillary sinus floor elevation with a lateral or transcrestal approach represent two surgical options to vertically enhance the available bone. Both augmentation techniques are clinically effective in achieving a vertical increase in crest dimension and are associated with high implant survival rates [4,6,28]. A re-analysis of data from a parallel-arm, randomized trial comparatively evaluating transcrestal sinus lift (tSFE) and lateral sinus lift (lSFE) was performed by Farina et al. (2023). Within each RBH interval (<4 mm or ≥4 mm), tSFE and lSFE groups were compared for chair time, surgery-related costs, morbidity, and radiographic parameters. Their results showed that at sites with RBH < 4 mm, pain was significantly higher in the tSFE group, while the lSFE group was associated with a significantly higher frequency of bruising. On the other hand, sites with RBH ≥ 4 mm showed a significantly lower frequency of postoperative signs and symptoms and less chair time was required in the tSFE group [29].

The factors that influence implant osteointegration in the posterior maxilla are bone quality, residual bone height, and the primary stability of the implants, with all of them being related [30].

Among the many factors mentioned, the ISQ value (implant stability coefficient) constitutes the mean unit of resonant frequency analysis (RFA) [31,32]. This parameter is crucial for the osseointegration of the implant. It is a mechanical process which depends on the characteristics of the bone where it is anchored. It is the result of the compressive stress generated into the bone during the insertion of the implant, in other words, the insertion torque of the implant is the measure of the frictional resistance that the implant encounters as it advances apically by means of a rotary movement on its axis [31,32]. Therefore, this method provides information on the quality of the bone at the implant placement site and the primary stability of the implant.

Regarding residual bone height, that seems to be a discriminating factor between the lateral or transcrestal approach. Tsai et al. [33] compared lateral and transcrestal approaches with four study groups; 1-stage bone-added osteotome sinus floor elevation procedure (BAOSFE) with simultaneous implant placement; 2-stage BAOSFE with delayed implant placement; 1-stage lateral window sinus floor elevation with simultaneous implant placement; and 2-stage lateral window sinus floor elevation with delayed implant placement. There was no significant difference in total bone height gain between the groups [33]. Farina et al. [29] have obtained similar outcomes in a randomized trial study comparing the transcrestal approach (tSFE) versus the lateral approach (LSFE), with simultaneous implant placement (21 treated with tSFE and 22 treated with lSFE), with a residual bone height of 3–6 mm. After a follow-up of 6 years, the implant survival rate was 100% [29].

Despite these promising results, protocols have not established the limits in terms of sufficient bone height for the transcrestal approach. Scientific evidence has demonstrated highly predictable results with the transcrestal approach when the available bone height is ≥5 mm [16,17,18]. Moreover, several clinical studies reported high implant survival rates with a residual bone height of ≤4 mm [14,23,24,33].

Gonzalez et al. [24] compared, in a multicenter study, sinus augmentation with a transcrestal approach in patients with a residual bone height ≤ 4 mm (group 1) versus those with >4 mm (group 2) of residual bone height, achieving a 100% success rate in group 1 (*n* = 35) and 98.51% success rate in group 2 (*n* = 67) after a mean follow-up period of 29.7 months.

The present study has shown that the tSFE procedure with OD and β-TCP resulted in a considerable vertical bone enhancement being observed at a twelve-month follow-up. The magnitude of these results paralleled previous studies on the same technique [25]. They evaluated the effectiveness and predictability of transcrestal elevation using osseodensification drills on 222 patients and 261 implants, obtaining favorable clinical results after a mean follow-up period of 35 months. Their records were similar to those obtained in our study: the mean residual bone height was 5.4 mm, obtaining a gain of 7 mm; in our study, the mean bone height was 5.7 mm, and a mean gain of 6.65 mm was obtained immediately after surgery and 5.75 mm at twelve months. A considerable bone gain with similar outcomes was obtained in previous investigations, where burs (CAS KIT, NeoBiotech, quokkaMED O.E. Athens, Greece) were used for tSFE [14,16]. However, when comparing the outcomes, the differences in the methods for assessing radiographic measurements could lead to bias. 

From the analysis of the mentioned studies, it seems that, despite different approaches for sinus floor elevation, studies are heterogeneous and there is a lack of consensus in terms of indications for the different techniques.

A variation proposed by some authors is the graftless tSFE technique, where the procedure is performed without the addition of a bone substitute. Following this variation, Andrés-García et al. [34] obtained a spontaneous bone gain of 3.86 mm 12 months after implant placement using osteotomes. Shalash et al. [35] also obtained similar results at 12 months in terms of implant survival when performing this same technique using Densah burs. The advantages of the graftless technique may be the reduction in economic costs and simplifying the surgical intervention. However, Cho et al. [19] conclude that adjunctive bone grafting is still indicated for cases requiring more than 2–3 mm of bone augmentation. Nevertheless, the evidence about the necessity of bone graft when residual bone height is below 5 mm is still controversial [22]. 

The graft’s volume contraction rate reported in the literature ranges from 20% to 50% for both autogenous bone and bone substitutes, such as demineralized freeze-dried bone allograft (DFDBA), mineralized freeze-dried bone allograft (FDBA), and xenografts. In more recent studies, resorbable bioceramics, made of a mixture of hydroxyapatite and beta-tricalcium phosphate, have gained popularity, demonstrating bioactivity and osteoconductive properties for vertical bone augmentation of the atrophied maxilla in different histological studies [36,37]. 

In the present study, beta-tricalcium-phosphate (*β-*TCP) putty was used as an alternative for the autogenous bone graft in sinus floor augmentation. As different studies have demonstrated, *β-*TCP is a suitable biomaterial to be used in the formation of new bone in sinus floor elevation procedures in humans, not only from the histomorphometric point of view, but also regarding the cellular and vascular quality of the regenerated bone [38,39].

A prospective case series study was performed by Tallarico et al. [40]. A total of 18 participants underwent transcrestal elevation of the sinus membrane and a total of 21 implants were placed. The mean residual bone height was 4.78 ± 0.88, with a bone gain of 12.78 ± 2.18 mm, which was similar to the bone gain obtained in our results (12.36 ± 1.20 mm). This study used a flowable synthetic bone substitute (biphasic calcium phosphate) composed of 60% (TCP) and 40% (HA), obtaining a physiologic contraction of 0.33 ± 0.29 mm after a 6-month follow-up, unlike that recorded in our results (0.90 ± 0.49 mm) [40]. 

Based on the available literature, β-TCP seems to be a promising graft material for sinus floor augmentation, and it may lead to new bone formation and substantial enhancement of bone height, better postoperative outcomes and fewer complications [40,41,42]. Nevertheless, more randomized clinical trials, with long follow-up periods and more homogenous studies in terms of the different techniques for sinus floor augmentation, are necessary to assess its long-term behavior. 

Primary stability in a dental implant is an essential factor for successful osseointegration. Surgical procedures and bone quality are some of the most common factors affecting primary stability. Achieving high insertion torque is also critical for primary stability [43,44]. This study investigated approaches to improve primary stability using an osseodensification technique, which was compared to the conventional under-drilling method used for low-density bones. They affirmed the OD technique improved stability in low-density bones (based on torque and RFA measurements), which could explain our study results. When using the osseodensification technique for transcrestal sinus lift, there was no statistical evidence for an association between primary stability and the bone height available at the different times of the study. Future studies, however, are needed to confirm this consideration. This relatively new concept with a universally compatible drill has been proposed to improve bone densification and transcrestal sinus lift. This procedure has also shown better osteotomy than conventional implant drills and it allows bone expansion in different bone densities [45]. These hypotheses we confirmed by a literature review by Pai et al. [46], where a total of 195 articles were collected and submitted for screening using inclusion and exclusion criteria. Other authors have evaluated the medium-term success of implants placed using transcrestal sinus lift with the under-drilling protocol and investigated the relationship between bone height and primary stability [47]. For 106 registered patients who received 253 implants, after 5 years of follow-up, no significant differences were found in the success rate of implants placed in RBH < 4 mm and those with higher bone height. Predictable results can be achieved by under-drilling, even in atrophic alveolar crests and with low bone quality. 

Long-term follow-up and histological examinations need to be performed to investigate the bone quality and stability of the apical part of the graft in association with the OD technique and the use of *β-*TCP as it relates to the possible positive correlation between insertion torque and primary stability with the bone gain. 

In terms of the clinicians’ experience, although they were instructed about the sequence of surgical instruments, had a supervisor, and had been involved in research protocols on the tSFE procedure and OD [25], it may be hypothesized that there were potential differences in skills as well as levels of experience, which could be one of the bias of imprecision of the study. Nevertheless, no information from previous studies is currently available on the impact of the operator’s experience on the outcomes of the tSFE procedure.

Due to the small sample of the study, further studies with a larger sample will be required to determine the statistical effectiveness in relation to the vertical gain and success rate of the OD technique with simultaneous placement of implants.

## 5. Conclusions

Within the limitations of this study, the use of osseodensification drills in combination with beta-tricalcium phosphate for transcrestal sinus lift may represent a less invasive alternative to direct sinus lift and for Summers osteotome technique, which is associated with a high success rate. This technique enabled vertical bone augmentation of up to 6.65 mm immediately after surgery, with a subsequent biological contraction of 0.90 mm. In addition, the implant insertion in atrophic patients was achieved maintaining a correct insertion torque and a correct primary stability. Nevertheless, it must be considered that these results refer to a pilot, non-randomized clinical trial. Consequently, randomized clinical studies with a larger sample of patients are recommended to confirm the results obtained.

## Figures and Tables

**Table 1:** Data collected on the patients included in the study.

		Count	Percentage
Gender	Males	4	30.77%
	Females	9	69.23%
Age (years)	≥30 years	0	0.00%
	31 to 60 years	9	69.23%
	>60 years	4	30.77%
Presence of diseases	NO	6	46.15%
	YES	7	53.85%

**Table 2:** Aspects related to surgical procedure.

Number	Single	4	30.77%
	Multiple	9	69.23%
Tooth to be rehabilitated	First premolar	4	30.77%
	Second premolar	0	0.00%
	First molar	7	53.85%
	Second molar	2	15.38%
Location	Implant between tooth	4	30.77%
	Implant to free end	9	69.23%
Bone type	D1	0	0.00%
	D2	0	0.00%
	D3	8	61.54%
	D4	5	38.46%
Tooth loss	>6 months	8	61.54%
	<6 months	5	38.46%
Drilling protocol	2.3 mm	0	0.00%
	3.0 mm	5	38.46%
	3.3 mm	8	61.54%
	4.0 mm	0	0.00%

**Table 3:** Results obtained in terms of the average height value at different times of the study.

	*N*	Mean	Standard Deviation	Typical Error	Confidence Interval for the Mean at 95%		Minimum	Maximum
					Upper Limit	Lower Limit		
RBH T0	13	5.70	1.57	0.44	4.75	6.65	3.47	8.44
RBH T1	13	12.36	1.99	0.55	11.15	13.56	9.10	16.09
RBH T2	13	11.45	1.55	0.43	10.52	12.39	8.40	13.98

**Table 4:** Related samples test (RBH).

	Related Differences					t	gl	*p*-Value
	Mean	Standard Deviation	Typical Error of the Mean	Confidence Interval for the Difference at 95%				
				Lower	Upper			
RBH T1–RBH T0	6.65	1.76	0.49	5.59	7.72	13.620	12	<0.001 ***
RBH T2–RBH T0	5.75	1.27	0.35	4.98	6.52	16.300	12	<0.001 ***
RBH T2–RBH T1	−0.90	0.82	0.23	−1.40	−0.41	−3.974	12	0.002 **

Significance level ** *p* < 0.01, and *** *p* < 0.001.

**Table 5:** Apical bone gain at T1 and T2.

	*N*	Mean	Standard Deviation	Typical Error of the Mean	Confidence Interval for the Difference at 95%		Maximum	Minimum
					Lower	Upper		
ABG T1	13	2.28	1.60	0.44	1.31	3.24	0.42	5.87
ABG T2	13	1.29	1.23	0.34	0.54	2.04	0.00	3.73

**Table 6:** Related samples test (ABG).

Related Samples Test								
	Related Differences					t	gl	*p*-Value
	Mean	Standard Deviation	Typical Error of the Mean	Confidence Interval for the Difference at 95%				
				Lower	Upper			
ABG T2–ABG T1	−0.99	0.72	0.20	−1.42	−0.55	−4.916	12	<0.001

**Table 7:** A bivariate correlation test was performed, and, thus, for the small size of the sample, the Spearman correlation test was used.

	Primary Stability (ISQ)			Insertion Torque (ISQ)	
Variable	*N*	Correlation Coefficient	*p*-Value	Correlation Coefficient	*p*-Value
RBH T1	13	0.123	0.689	−0.163	0.595
RBH T2	13	0.049	0.873	0.267	0.378
ABG T1	13	0.242	0.425	0.211	0.488

## Data Availability

The original contributions presented in the study are included in the article, further inquiries can be directed to the corresponding authors.
